# Expression of lncRNA LINC00943 in lung squamous cell carcinoma and its relationship with tumor progression

**DOI:** 10.1186/s13019-024-02771-2

**Published:** 2024-04-16

**Authors:** Zhenshan Zhao, Haiyang Li, Jing Li, Yao Rong, Lidong Zhao, Menghui Hao, Faming Tian

**Affiliations:** 1 Department of Thoracic Surgery, KaiLuan General Hospital, Tangshan, 063000 Hebei China; 2 Department of Oncology, KaiLuan General Hospital, No. 57, Xinhua East Road, Tangshan, 063000 Hebei China; 3 Department of Clinical Laboratory, TangShan GongRen Hospital, Tangshan, 063003 Hebei China; 4 Department of College of Basic Medical Sciences, North China University of Science and Technology, Tangshan, 063210 Hebei China

**Keywords:** LUSC, LINC00943, miR-196b-5p, Prognosis, Migration, Invasion

## Abstract

**Background:**

Molecular biology has been applied to the diagnosis, prognosis and treatment of various diseases, and long noncoding RNA LINC00943 (lncRNA LINC00943; LINC00943) plays an important role in a variety of cancers. Therefore, this study explored the prognostic role of LINC00943 in lung squamous cell carcinoma (LUSC) and understood its impact on the development of LUSC.

**Methods:**

There are 89 LUSC patients were involved in current assay. By detecting the expression of LINC00943 and miR-196b-5p in tissues and cells, LINC00943 and its correlation with the characteristics of clinical data were analyzed. The biological function of LINC00943 was studied by Transwell migration and invasion assays. In addition, Pearson correlation coefficient and luciferase activity experiments were chosen to characterize the relationship between LINC00943 and miR-196b-5p and explore the mechanism of LINC00943.

**Results:**

Compared with normal controls, LINC00943 expression in LUSC tissues and cells was significantly reduced, miR-196b-5p was markedly increased, there was a negative correlation between LINC00943 and miR-196b-5p. According to the in vitro cell experiments, migration and invasion of LUSC cells were suppressed by overexpression of LINC00943. Besides, LINC00943 was demonstrated to have prognostic power and targeting miR-196b-5p was involved in the progression of LUSC.

**Conclusions:**

Overexpression of LINC00943 was molecular sponge for miR-196b-5p that controlled the deterioration of LUSC, which had great potential as a prognostic biomarker for LUSC.

## Background

Lung squamous cell carcinoma (LUSC) is the second most common type of lung cancer after lung adenocarcinoma, with the highest morbidity and mortality in China, and the number of patients continues to increase [1, 2]. The patient eventually mutated into LUSC due to chronic stimulation and damage to the columnar epithelial cells of the bronchial mucosa, loss of cilia, and squamous metaplasia of basal cells [3]. The specific etiology of LUSC has not yet been determined, but smoking, air pollution, family inheritance and bad living habits are all high-risk factors for the disease [4]. Surveys have shown that surgical treatment, radiotherapy and chemotherapy can relieve symptoms and prolong life. However, the recurrence rate of patients is extremely high, the prognosis is poor, and the survival rate of approximately 60% [5]. Therefore, identifying high-risk groups with poor prognosis through prognostic factors as early as possible is of great practical significance for the treatment of LUSC and the improvement of patient survival rates.

For the treatment of LUSC patients, in addition to the commonly used surgical resection, radiotherapy and chemotherapy, and drug therapy, molecular therapy represented by lncRNAs has also received attention in recent decades [6]. For example, lncRNA IGKJ2-MALLP2 suppressed reproduction and angiogenesis of LUSC cells [7]. LncRNA AC026166.2-001 and RP11-169D4.1-001 have clinical significance in the treatment of LUSC patients, which can be used as independent prognostic factors for patient survival [8]. LncRNA HAGLROS has also been shown to accelerate the growth of LUSC cells through multiple signaling pathways [9]. Recent studies have found that lncRNAs coordinate with microRNAs (miRNAs) and protein-coding mRNAs to regulate the progress of lung cancer. Ni et al. demonstrated that the lncRNA-SOX2OT/miRNA-194-5p/RAC1 signaling axis synergistically promotes the metastasis of non-small cell lung cancer (NSCLC) [10]. Xia et al. promoted the development of lung cancer by absorbing different miRNAs through LINC01140, providing a new ideal target for tumor therapy [11].

Moreover, LINC00943 was found to be involved in the regulation of Parkinson’s disease [12], gastric cancer [13], clear cell renal cell carcinoma [14] and breast cancer [15] in the existing literature. However, the expression and function of LINC00943 in LUSC, and whether it can play a role in affecting tumor progression have not been documented. This experiment focused on exploring the potential of LINC00943 as a prognostic biomarker in LUSC and to understand the mechanism of LINC00943 binding to miRNA in LUSC.

## Methods

### Collection of tissue specimens

LUSC tissue specimens confirmed by professional pathologists were collected in KaiLuan General Hospital from 2015 to 2016. The 89 LUSC patients who confirmed to participate in this study did not receive any anti-tumor treatment before sampling, while patients with multiple diseases were excluded. The LUSC tissues and adjacent normal tissues removed from the patients were immediately frozen in liquid nitrogen and stored in a refrigerator at -80°C for subsequent experiments.

This study was performed in line with the principles of the Declaration of Helsinki. Approval was granted by the Ethics Committee of KaiLuan General Hospital approved this study, and all participating patients signed an informed consent form as required. The clinical indicators of LUSC patients, such as age, gender, tumor size, and differentiation are summarized in Table 1. All LUSC patients enrolled in the study were followed for 5 consecutive years by face-to-face interview and telephone communication, and survival information was recorded.

**Table 1:** Correlation of the lncRNA LINC00943 expression with clinical characteristics in LSCC

Parameters	Cases (*n* = 89)	LINC00943 expression		*P*
		Low (*n* = 45)	High (*n* = 44)	
Age				0.593
≤ 60	46	22	24	
> 60	43	23	20	
Gender				0.460
Male	43	20	23	
Female	46	25	21	
Tumor size				0.161
≤ 5 cm	50	22	28	
> 5 cm	39	23	16	
Differentiation				0.071
Well, Moderate	46	19	27	
Poor	43	26	17	
Lymph node metastasis				0.025
Negative	38	14	24	
Positive	51	31	20	
TNM stage				0.012
I, II	31	10	21	
III, IV	58	35	23	

### Cell lines and transfection

Four human LUSC cell lines (H520, H1703, EBC-1, H2170) and human bronchial epithelial cells BEAS2B were derived from the Shanghai Cell Bank of the Chinese Academy of Sciences. All cells were grown in 6-well cell culture plates in RPMI 1640 medium (Gibco/Life Technologies, USA), which contains 10% fetal bovine serum (FBS; Gibco, USA) and 1% MEM non-essential amino acids (MEM NEAA; Gibco, USA) at 37°C in a 5% CO_2_ incubator.

The overexpression vector plasmid pcDNA3.1 required for transfection was derived from GenePharma Co. Ltd. (Shanghai, China). In the transfection experiment, the constructed plasmid overexpressing LINC00943 (pcDNA3.1-LINC00943) was transferred into H520 and H2170 cells using Lipofectamine 3000 reagent (Invitrogen, USA), and the untreated cells were regarded as the control group.

### Real-time quantitative PCR assay

The TRIZOL (Sigma-Aldrich) method was chosen to obtain total RNA, and precipitate and wash RNA with 85% ethanol to obtain miRNAs. Precision nanoScript2 Reverse Transcription Kit (Primerdesign) and miRNA 1st Strand cDNA Synthesis Kit (Vazyme) were selected to reverse transcription to synthesize cDNA (A260/A280 ratio: between 1.8 and 2.0) for lncRNA and miRNA respectively. The reaction system was configured using ChamQ SYBR qPCR Green Master Mix and miRNA Universal SYBR qPCR Master Mix (Vazyme), and RT-qPCR detection was performed on a 7500 Real-Time PCR system. Cycling parameters: incubation at 95°C for 10 min, followed by 40 cycles of 95°C for 15 s, 60°C for 60 s, and 72°C for 15 s, and finally extension at 72°C for 10 min. The endogenous control of LINC00943 and miR-196b-5p utilized glyceraldehyde-3-phosphate dehydrogenase (GAPDH) and U6, while the LINC00943 and miR-196b-5p expression were calculated by the 2^−ΔΔCt^ method. The primer sequences are as follows: LINC00943, F-5’-GATGAACCACCCATGGCCT-3’; R-5’-CTTCCAGGAATGGAAGCCA-3’. miR-196b-5p, F-5’-ATCCTTCCTAGTCCAGCC-3’; R-5’-ACCTGGCGGCACTCCTTA-3’.

### Transwell assay

The number of LUSC cells migration and invasion was detected by Transwell assay, and the steps were similar, both of which were performed in a 24-well Transwell chamber. The specific steps are as follows: first add RPMI-1640 medium in which the transfected cells (2 × 10^4^ cells/well) have been suspended in the upper chamber; then add 10% FBS and RPMI-1640 medium to the lower chamber to induce cell migration; 24 h later, use 4% paraformaldehyde was fixed for 30 min to migrate cells in the lower chamber, then the cells were washed with PBS; finally, stained with 0.1% crystal violet for 20 min at room temperature. It should be noted that in cell invasion assay, the upper chamber needs to be coated with Matrigel (200 mg/ml, BD Biosciences, Franklin Lakes, NJ, USA) 30 min in advance at 37°C. Under the optical microscope, observe the number of cells in five random fields of view.

### Luciferase reporter assay

The luciferase reporter vector pmirGLO (Promega, Shanghai, China) was selected to construct WT-LINC00943 (wild-type) and MUT-LINC00943 (mutant-type). The H520 cells were seeded in 24-well plates and respectively co-transfected with miR-196b-5p mimic, miR-196b-5p inhibitor, mimic NC, and inhibitor NC with the help of Lipofectamine 3000 reagent. After 48 h, the measurement was performed by a dual-luciferase reporter assay kit (Promega) following the manufacturer’s protocol.

### Statistical analysis

The statistical analysis was analyzed via the SPSS 20.0 software (SPSS, USA) and GraphPad Prism 5.0 software (GraphPad Software, USA). All experimental data are expressed as mean ± standard deviation (SD) deviation, and the correlation between the expression of LINC00943 and the clinical data of patients was compared by the χ^2^ test. The prognostic significance of LINC00943 can be analyzed by Kaplan-Meier and Cox regression analysis. *P* value lower than 0.05 is considered statistically significant. Each experiment was repeated at least three times.

## Results

### Expression of LINC00943 in LUSC tissues

**Fig. 1:**
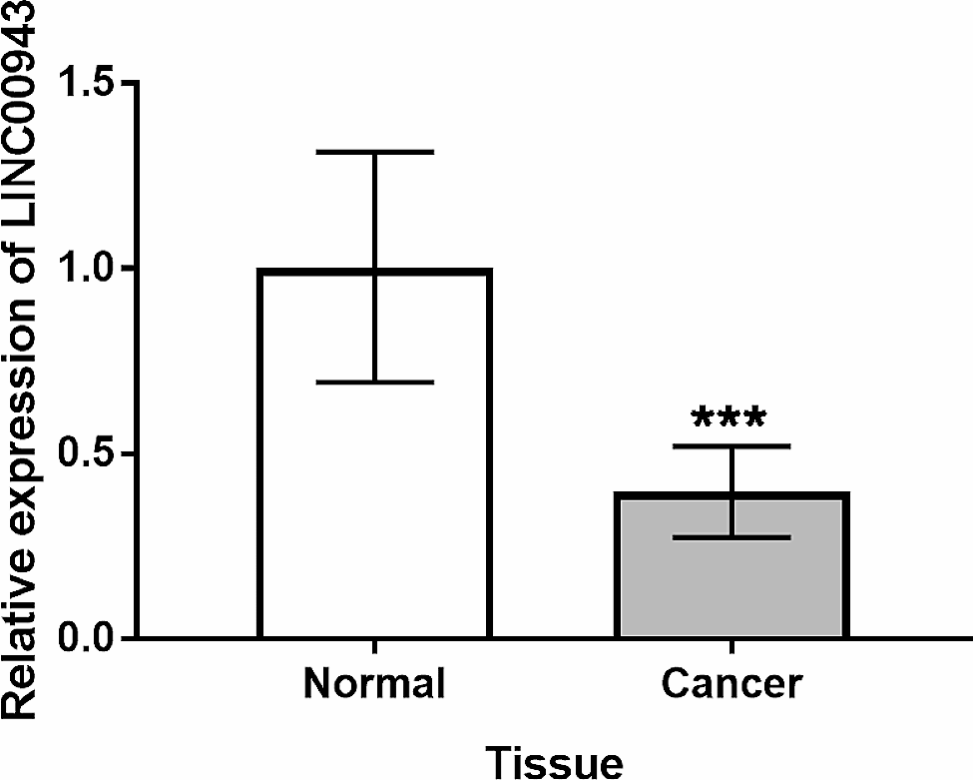
LINC00943 was downregulated in LUSC tissues by RT-qPCR detection. ***P < 0.001

LINC00943 level in tumor tissues and normal tissues was detected via RT-qPCR. As exhibited in Fig. 1, LINC00943 were significantly reduced in tumor tissues, compared with adjacent normal tissues. According to the average expression of LINC00943, LUSC patients were divided into high expression group (*n* = 44) and low expression group (*n* = 45), and the correlation between LINC00943 expression and clinical characteristics of patients was analyzed (Table 1). The data showed that lymph node metastasis (*P* = 0.025) and TNM stage (*P* = 0.012) were associated with low expression of LINC00943.

### Prognostic potential of LINC00943 in LUSC

**Fig. 2:**
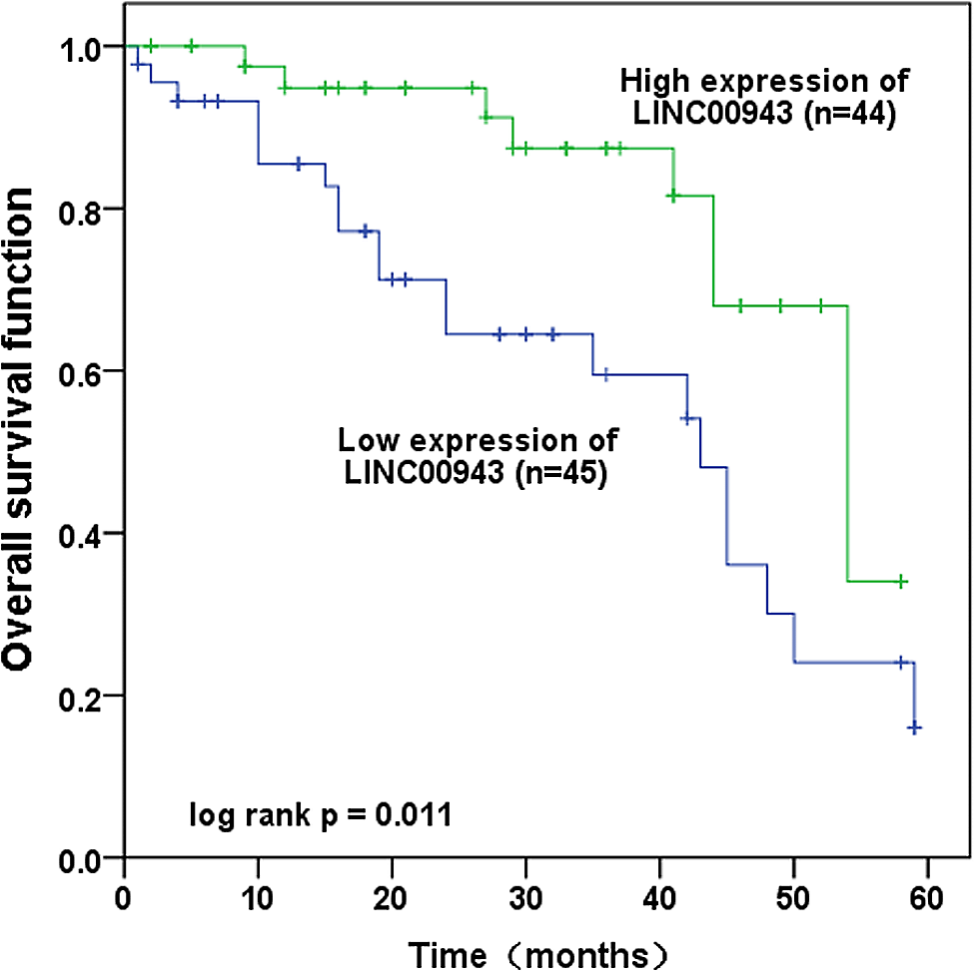
The survival probability of LUSC patients with high or low expression of LINC00943 within five years was analyzed by Kaplan-Meier method. The overall survival rate of patients with low expression of LINC00943 was lower than that of patients with high expression (log-rank test *P* = 0.011)

The prognostic value of LINC00943 in LUSC was evaluated by Kaplan-Meier curve analysis and log-rank test. According to the analysis of LINC00943 expression and progression-free survival probability, the survival probability of high LINC00943 expression group (*n* = 44) was higher than that of low LINC00943 expression group (*n* = 45) in five years (Fig. 2). Additionally, Table 2 illustrated that LINC00943 (HR = 4.007, 95% CI = 1.568–10.237, *P* = 0.004), differentiation (HR = 2.805, 95% CI = 1.180–6.671, *P* = 0.020), lymph node metastasis (HR = 3.241, 95% CI = 1.384–7.591, *P* = 0.007), and TNM stage (HR = 3.874, 95% CI = 1.267–11.845, *P* = 0.018) were independent prognostic factors for five-year overall survival in LUSC after multivariate Cox analysis.

**Table 2:** Multivariate Cox analysis of clinical characteristics in relation to overall survival

Parameters	Multivariate analysis		
	HR	95% CI	*P*
LncRNA LINC00943	4.007	1.568–10.237	0.004
Age	1.107	0.484–2.534	0.809
Gender	1.365	0.638– 2.922	0.423
Tumor size	2.456	0.903–6.683	0.078
Differentiation	2.805	1.180–6.671	0.020
Lymph node metastasis	3.241	1.384–7.591	0.007
TNM stage	3.874	1.267– 11.845	0.018

### Assay in vitro of LUSC cells

**Fig. 3:**
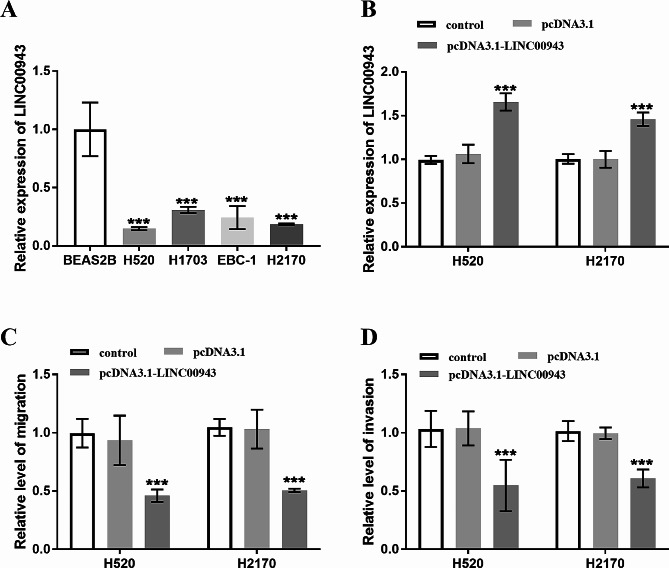
The effect of overexpression of LINC00943 on LUSC cells was analyzed in vitro assays. (A) LINC00943 level in four LUSC cell lines (H520, H1703, EBC-1, H2170) and normal cell BEAS2B was detected. (B) LINC00943 expression ensured the transfection efficiency. (C) and (D) Migratory level and invasive ability of H520 and H2170 cells were measured via Transwell assay. ***P < 0.001

To further confirm the expression of LINC00943 in cells, Fig. 3A showed downregulation of LINC00943 detected by RT-qPCR compared to human bronchial epithelial cells BEAS2B in LUSC cell lines (H520, H1703, EBC-1, H2170). Based on the relatively lower expression of LINC00943 in H520 and H2170 cells, all of them were chosen for subsequent experiments. The overexpression pcDNA3.1-LINC00943 was constructed with pcDNA3.1 as the vector, LINC00943 expression in H520 and H2170 cells was detected, and the function and influence of LINC00943 in LUSC were studied. In Fig. 3B, LINC00943 was obviously increased in H520 and H2170 cells after transfection. Moreover, as displayed in Fig. 3C and D, Transwell assay results demonstrated that pcDNA3.1-LINC00943 suppressed the migratory level and invasive abilities of H520 and H2170 cells. That is, LINC00943 overexpression may alleviate the exacerbation of LUSC.

### LINC00943 interacted with miR-196b-5p

**Fig. 4:**
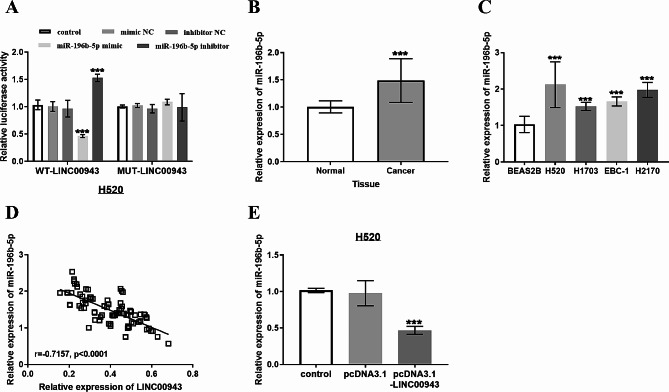
Interaction of LINC00943 and miR-196b-5p. (A) Luciferase activity was examined in H520 cell co-transfected with miR-196b-5p mimic, miR-196b-5p inhibitor, mimic NC or inhibitor NC and WT-LINC00943 or MUT-LINC00943. (B) and (C) Compared with normal tissues adjacent to cancer, miR-196b-5p expression was up-regulated in tumor tissues and cells. (D) The reverse correlation between LINC00943 and miR-196b-5p expression (*r* = -0.7157, *P* < 0.0001). (E) Overexpression of LINC00943 inhibited the expression of miR-196b-5p in H520 cell. ***P < 0.001

Online prediction and bioinformatics analysis revealed that LINC00943 and miR-196b-5p form multiple base pairs. According to the luciferase assay showed that miR-196b-5p mimic significantly reduced the luciferase activity of cells transfected with WT-LINC00943, miR-485-5p inhibitor increased luciferase activity, whereas luciferase activity was not affected in MUT-LINC00943 transfected H520 cells (Fig. 4A). The content of miR-196b-5p was tested, and the increased expression of miR-196b-5p in LUSC tissues and cells was shown in Fig. 4B and C. Figure 4D characterizes the negative correlation between LINC00943 and miR-196b-5p through the Pearson correlation coefficient, indicating that high expression of LINC00943 inhibited the expression of miR-196b-5p (*r* = -0.7157, *P* < 0.0001). Simultaneously, the relative expression of miR-196b-5p in H520 cells after transfection with pcDNA3.1-LINC00943 elaborated that the high expression of LINC00943 inhibited the expression of miR-196b-5p (Fig. 4E).

## Discussion

LUSC is a unique subtype of NSCLC that accounts for 25% to 30% of lung cancers [16, 17]. LUSC mostly occurs in elderly men who are addicted to smoking and is greatly affected by personal living habits [18, 19]. Most patients have no significant symptoms in the early stage, or are accompanied by symptoms such as cough, bloody sputum, chest pain, and fever [20]. With the gradual deterioration of the disease will appear corresponding respiratory symptoms or cancer metastasis expansion. Traditional surgery and drug therapy have certain limitations, so for better treatment and prognosis, researchers focus on the research and evaluation of molecular biomarkers. In the past few years, many studies have shown that lncRNAs play important role in tumor growth and metastasis.

Li et al. stated that under-expression of STARD13-AS in LUSC that affected tumor cells growth and motility [21]. In the research of Yin et al., it was found that the decrease of SFTA1P expression was related to the occurrence and metastasis of LUSC and was expected to become a new prognostic target for LUSC [22]. Here, we demonstrated that LINC00943 was lowly expressed in LUSC tissues and cells and proved to be a prognostic factor for LUSC, which was similar to the existing evidence. In addition, the significantly high expression of LINC00943 in this study negatively affected the migration and invasion of tumor cells, which was consistent with Wan et al. ‘s detection that overexpression of WT1-AS reduced the migration and invasion of NSCLC cells [23]. It is inferred that LINC00943 may regulate the occurrence and development of LUSC.

It has been reported that miRNAs, as conserved non-coding single-stranded RNA molecules, are involved in a variety of physiological and pathological processes [24]. As a sponge of miRNAs, lncRNA forms the corresponding lncRNA/miRNA axis to regulate transcription and tumor cell proliferation [25, 26]. This study predicted that LINC00943 and miR-196b-5p have binding sites. Liang et al. learned that miR-196b-5p mediates TSPAN12 and GATA6 factors to accelerate the development of NSCLC [27]. The downregulation of RSPO2 by miR-196b-5p also provided a new theoretical basis for the treatment of lung adenocarcinoma [28]. In addition, studies on miR-196b-5p elucidated that miR-196b-5p targets and negatively regulates NFKBIA to accelerate the growth of NSCLC tumor cells [29]. Existing evidence also described the differential expression and regulation of miR-196b-5p in diseases such as medulloblastoma, keloid tumor, infantile hemangioma [30–32]. We proved that miR-196b-5p was upregulated in LUSC, that LINC00943 directly targets miR-196b-5p to mediate disease in LUSC patients, and that LINC00943 and miR-196b-5p are negatively correlated. These results indicate that LINC00943/miR-196b-5p axis plays a regulatory role in LUSC, suggesting a new therapeutic target.

In particular, the process design of this study is relatively complete, but the lack of in vivo cell experiments limits the clinical application and promotion of LINC00943 as a prognostic marker. In addition, it is necessary to recruit more volunteers to participate in our subsequent research to improve the persuasiveness of the research results. There is still a long way to go before theoretical research can actually be applied to clinical experiments.

## Conclusions

In summary, all data and results indicate that LINC00943 was downregulated and miR-196b-5p was significantly upregulated in LUSC tissues and cells. Overexpression of LINC00943 inhibited the progress of LUSC by negatively regulating miR-196b-5p. LINC00943 is promising to be a biomarker for the prognosis of LUSC.

## Data Availability

The datasets used and/or analysed during the current study are available from the corresponding author on reasonable request.
